# The Slow Depolarization Following Individual Spikes in Thin, Unmyelinated Axons in Mammalian Cortex

**DOI:** 10.3389/fncel.2019.00203

**Published:** 2019-05-16

**Authors:** Morten Raastad

**Affiliations:** Department of Physiology, School of Medicine, Emory University, Atlanta, GA, United States

**Keywords:** action potential, axon, unmyelinated axons, after-potentials, depolarizing after-potential, conduction failures, presynaptic

## Abstract

An important goal in neuroscience is to understand how neuronal excitability is controlled. Therefore, Gardner-Medwin's 1972 discovery, that cerebellar parallel fibers were more excitable up to 100 ms after individual action potentials, could have had great impact. If this long-lasting effect were due to intrinsic membrane mechanisms causing a depolarizing after-potential (DAP) this was an important finding. However, that hypothesis met resistance because the use of K^+^ sensitive electrodes showed that synchronous activation, as commonly used in excitability tests, increased extracellular K^+^ concentration sufficiently to explain much of the hyperexcitability. It is still controversial because intra-axonal recordings, which could have settled the debate, have not been made from parallel fibers or other axons of similar calibers. If it had not been for the fact that such thin axons are, by far, the most common axon type in cortical areas and control almost all glutamate release, it would be tempting to ignore them until an appropriate intra-axonal recording technique is invented. I will go through the literature that, taken together, supports the hypothesis that a DAP is an intrinsic membrane mechanism in cerebellar parallel fibers and hippocampal Schaffer collaterals. It is most likely due to a well-controlled process that stops the fast repolarization at a membrane potential positive to resting membrane potential, leaving the membrane more excitable for ~100 ms during a slow, passive discharge of the membrane capacitance. The DAP helps reduce failures but can also cause uncontrolled bursting if it is not properly controlled. The voltage at which the fast repolarization stops, and the DAP starts, is close the activation range of both Na^+^ and Ca^2+^ voltage activated channels and is therefore essential for neuronal function.

## Introduction

The after-potentials of the action potentials (“spikes”), either hyper- or de-polarizing relative to resting membrane potential, are excitability-controlling mechanisms. Such after-potentials have been extensively studied in regions near the soma where some of their functions are obvious: they reduce or increase the amount of current needed to reach threshold for spike initiation at the axon initial segment (AIS). In the axon, after the spike is triggered at the AIS, the functions of after-potentials are less obvious. That may be one of the reasons considerably less is known about after-potentials in axons, particularly in the very thin, typical cortical axons (TCAs).

Also, when spikes in TCAs have been studied the focus has been mostly on the fast rather than the slow part of the spike. This is because the shape of the fast spike has a well-established impact on transmitter release (Augustine, 1990; Sabatini and Regehr, 1997; Borst and Sakmann, 1999). In contrast, the study of effects of after-potentials on transmitter release is relatively new (Clarke et al., 2016; Sierksma and Borst, 2017), and their influences on conduction properties like speed, endurance, bursting, and failures, which are the main topics of this article, have received even less attention.

However, the most important reason for the lack of knowledge is that TCAs may be the worst preparations for investigating spike mechanisms because the tiny diameters of these axons (on average 0.17 μm, Westrum and Blackstad, 1962; Palay and Chan-Palay, 1974; Shepherd and Harris, 1998) have prevented the use of direct intracellular voltage recordings from intact axons. The revolution in genetic and molecular techniques has not solved this problem.

Unfortunately, we cannot get around the TCAs because they are by far the most common axon type in mammalian cortices (including hippocampal, cerebellar, and neo-cortex). Most of the brain's release of the excitatory transmitter glutamate is controlled by presynaptic “boutons” found along most of the paths of TCAs. They comprise the largest fractions of cortical volume (~50%) and cellular membranes (~60%) (Mishchenko et al., 2010) and are usually by far the largest part of neurons' volume and membrane surface (Braitenberg and Schüz, 1998; Wu et al., 2014).

The first data suggesting an after-potential in TCAs came from Gardner-Medwin in 1972 when he showed that cerebellar parallel fibers were more excitable up to 100 ms after individual action potentials. Although a depolarization after the spike was considered likely, the mechanism for this depolarization was suggested to be extracellular K^+^ accumulation due to the synchronized activation of many parallel axons. Many years later, Wigström and Gustafsson (1981) used almost identical tests of TCAs in hippocampal slices and found the same excitability changes but concluded that the post-spike hyperexcitability was due to an intrinsic mechanism of the axons. One of the main aims of this article is to detangle the excitability-increasing effects of changes in extracellular K^+^, and on the other hand the axon-intrinsic mechanism that creates a depolarizing after-potential (DAP) in TCAs.

Despite the limited opportunity to record intra-axonal potentials, it has over many years from a variety of technical approaches accumulated important data on the TCA spike, its propagation, and the DAP. Because of the slow accumulation of data some reports may have gone unnoticed, and this review will therefore summarize and synthesize knowledge about the TCA spike and their DAP. Almost all data are from cerebellar parallel fibers and hippocampal Schaffer collaterals, but I will refer to them as “TCAs,” for convenience, but also as a reminder that they a similar to most axons in cortical regions with respect to calibers, boutons, and transmitter type. Also those pyramidal neurons that have myelin around their proximal axon usually end up as thin terminal axons that fit the description of a TCA, although their spike or after-potentials have not been investigated. We must therefore keep in mind that axons other than Schaffer collaterals and parallel fibers may be different.

I will focus on the DAP in the context of excitability control and spike propagation in TCAs, and mostly ignore the TCAs' transmitter releasing function which is covered elsewhere (for example (Debanne et al., 2011; Fioravante and Regehr, 2011)).

The sum of the available information strongly suggests that that hippocampal and cerebellar thin axons, examples of TCAs, have two distinct phases of repolarization; one fast that stops relatively abruptly at a well-controlled membrane potential more positive than the resting membrane potential, and thereafter the DAP which is a slow, passive decay back to resting membrane potential.

## TCA Post-Spike Hyperexcitability—an Axonal Membrane Mechanism or the Result of Unspecific Increase in Extracellular K^+^ Concentration?

### Hyperexcitability

**Figure 1:**
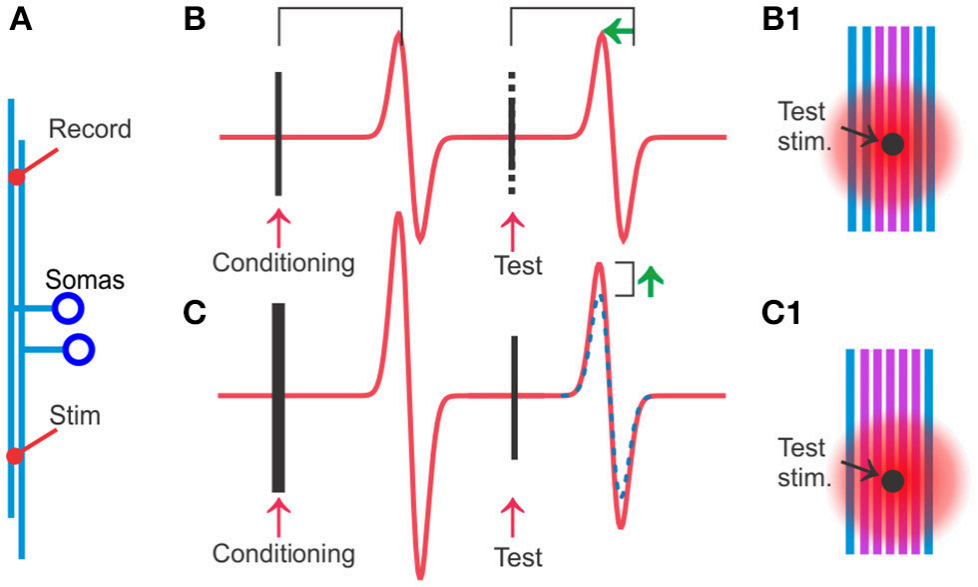
Hyperexcitability specific to the activated axons can be detected and quantified by using conditioning and test stimuli on populations of TCAs. **(A)** Stimulation and recording electrodes are positioned typically 2–3 mm apart among the cerebellar parallel fibers or other TCAs. **(B)** A test stimulus is given 20 ms after the conditioning stimulus. When conditioning and test stimuli have equal strength, the test stimulus will activate the same axons as the conditioning stimulus and therefore the two spike population responses have similar amplitudes. However, the response to the test stimulus occurs at a shorter latency (green arrow) because the conditioned axons (violet in **B1**) are more excitable and the spike travels faster. This hyperexcitability can be quantified by reducing the stimulus strength of the test stimulus (dotted part of the stimulus artifact). At moderate reductions, typically 15–40%, the test response keeps its original amplitude. The interpretation is that the recently conditioned axons (violet in **B1**) have lower activation threshold, and even the reduced test stimulus is sufficient to activate all the violet, conditioned axons. **(C)** When the conditioning stimulus is stronger than the test (more violet, activated axons in **C1** than in **B1**), but the test strength is the same as in **(B)**, the test response will get a larger amplitude than in **(B)** (compare with dotted blue which is response to conditioning and test stimulus of equal strength). The interpretation is that the stronger conditioning stimulus leaves more axons with reduced threshold in **(C)** than in **(B)** (violet in **B1**) allowing the test stimulus in **(C)** to activate more axons than in **(B)**.

The hyperexcitability that follows individual spikes in TCAs, which was the first hint of a DAP in such axons, can be reliably demonstrated through its effects on population spike amplitude and propagation speed (explained in Figure 1). These effects of a single conditioning stimulus were first shown by Gardner-Medwin (1972) in the cerebellar parallel fibers of the anesthetized cat, and by Wigström and Gustafsson (1981) in the Schaffer collaterals in hippocampal slices. Gardner-Medwin (1972) concluded that “The apparent increase of the conduction velocity of the parallel fibres is so marked that it distinguishes them from all the other fibres in the mammalian nervous system in which the after-effects of stimulation have been studied.”

Both these studies found the maximal hyperexcitable effect around 20–30 ms after an electrical activation of the spikes, and they quantified the increase in excitability to 20–40% by studying how much they could reduce the strength of a second stimulus without losing any amplitude of the population spike amplitude. The similarity of these results, despite the large difference in the preparations, suggests that the hyperexcitability is a robust phenomenon.

### Hyperexcitability in Peripheral Axons

Excitability of *peripheral* axons in mammals and many other species has been extensively studied by recording changes in conduction speed. Particularly the thin, unmyelinated C-fibers have pronounced activity-dependent changes in conduction speed and may therefore help us understand mechanisms in TCAs. Many of these investigations use trains of stimuli at high frequency which activate many mechanisms, for example hyperpolarization due to the Na-K-pump (Ritchie and Straub, 1957) and depolarization due to increasing extracellular K^+^ (Frankenhaeuser and Hodgkin, 1956). These factors can lead to periods of speeding, slowing and failures. Because of the difficulty in interpreting such mixtures of mechanisms, I will focus on the excitability changes that occur after single spikes (“post-spike hyperexcitability”).

Many peripheral axon types show post-spike hyperexcitability, with duration similar to or sometimes longer than that observed in Schaffer collaterals and parallel fibers. Human sympathetic C-fibers have a hyperexcitable period of ~1,000 ms (Bostock et al., 2003). Because of the long conduction distances that can be investigated in humans, the speeding-up due to this hyperexcitability can be considerable. For example, in human C-fibers of mechano- and heat-insensitive type, two spikes triggered 50 ms apart caught up >65% of that interval over ~500 mm conduction distance (Weidner et al., 2003). Most increases after single spikes in humans are, however, small (often <5%), possibly due to masking of some of the effect by parts of the axon with different activity-dependent properties (discussed in Bostock et al., 2003). Myelinated axons have a DAP with an underlying mechanism that relies on a high-resistance pathway between the cell membrane and the myelin sheet (Barrett and Barrett, 1982) and is therefore not directly similar to the DAP in TCAs.

To summarize, increases in population propagation speed and amplitude are robustly demonstrated in whole animals (Gardner-Medwin, 1972), cerebellar and hippocampal slices (Wigström and Gustafsson, 1981), at temperatures between 22 and 35°C (Soleng et al., 2004; Pekala et al., 2016), and in peripheral cold-sensitive C-fibers and sympathetic axon in humans (Bostock et al., 2003).

### Does Extracellular Potassium Contribute to, or Even Fully Explain the Hyperexcitability?

Although there has not been doubt about the existence of the hyperexcitability in TCAs, there has been considerable uncertainty about its cause. The most likely causes are that the spike is followed by a depolarization due to (a) increased extracellular potassium concentration ([K^+^]_o_), or (b) an intrinsic membrane mechanism. This distinction is important because electrical activation synchronizing many axons may give [K^+^]_o_ increases (Δ[K^+^]_o_) never occurring during normal brain activity, while an intrinsic membrane mechanism is likely to be important for normal axonal function.

Around the time Gardner-Medwin published his excitability-increasing effects there was an emerging awareness of the excitability-modifying effects of neurons' K^+^ efflux and accumulation in extracellular space (Frankenhaeuser and Hodgkin, 1956). Even individual bursting Purkinje cells could produce Δ[K^+^]_o_ of ~1 mM in their vicinity (in turtle, Hounsgaard and Nicholson, 1983). Much of the magnitude and time-course of the excitability increase seen with *repetitive* extracellular electrical stimulation could be explained by [K^+^]_o_ recorded by K^+^-sensitive electrodes (Malenka et al., 1981, 1983; Kocsis et al., 1983), but the critical question of whether *single* spikes could raise [K^+^]_o_ enough to explain the large (15–20%) increase in conduction speed was not addressed.

If we as a starting point assume that the excitability-increasing effect of a fast rise in Δ[K^+^]_o_ in response to electrical activation of axons is similar to the effect of a slower increase in Δ[K^+^]_o_ achieved by increasing the bath concentration of K^+^, the post-spike hyperexcitability cannot be explained by Δ[K^+^]_o_. Estimated Δ[K^+^]_o_ per stimulus in bundles of simultaneously activated unmyelinated axons in the brain is 0.2–0.5 mM (Fritz and Gardner-Medwin, 1976; Kocsis et al., 1983; Aitken and Somjen, 1986). In contrast, raising bath [K^+^]_o_ from 2.5 to 4 mM increased conduction speed by only 4.6% (Kocsis et al., 1983; Soleng et al., 2004). So, a [K^+^]_o_ 3–6 times higher than the single-stimulus concentration gives only 1/3 to ¼ of the speed increase caused by individual spikes.

However, fast stimulus induced Δ[K^+^]_o_ may have larger effects on excitability that the slower bath-applied Δ[K^+^]_o_ because with slow increase membrane stabilizing mechanisms like the H-current and the Na-K-pump will have time to counteract the depolarizing effect of Δ[K^+^]_o._ This idea may be possible to test because postsynaptic structures contribute significantly to the stimulus-induced Δ[K^+^]_o_. Around 50% reduction of Δ[K^+^]_o_ was reported when synaptic signals were blocked by low Ca^2+^ and high Mg^2+^, or Mn^2+^ in hippocampal slices, cerebellum *in vivo*, and dorsal horn of rat spinal cord slices (Nicholson et al., 1978; Urbán et al., 1985; Aitken and Somjen, 1986). One publication reported that post-spike hyperexcitability (speed) was not changed after block of synaptic signals with adenosine (Kocsis et al., 1984). If also adenosine-block of synaptic signals reduces Δ[K^+^]_o_, that finding would suggest that Δ[K^+^]_o_ is not responsible for all of the post-spike hyperexcitability.

A third argument against K^+^ as a major factor is obtained by reducing Δ[K^+^]_o_ around the tested axon by spatially separating the conditioning and testing stimulus. This was done by using different branches of the same neuron for conditioning and test stimulus (Soleng et al., 2004). The threshold reduction obtained by this approach were similar to that of cerebellar axons in whole animals (14% reduction at 34°C and 30 ms interval).

### Single Axon

The critical experiment to test if the hyperexcitability is specific to the activated axon is of course to measure the excitability when only one axon is activated. This was done on cerebellar parallel fibers with a somatic tight-seal electrode triggering a somatic spike and a separate electrode testing the threshold for spike activation of the axon (Palani et al., 2012). In this configuration the threshold reduction and its time course (maximal threshold reduction 25% at 20 ms after a spike) was similar to the post-spike hyperexcitability described for parallel fibers in whole animals (Gardner-Medwin, 1972), supporting the idea that the post-spike hyperexcitability is an intrinsic property of the parallel fibers.

### How Much Does the Combined K^+^ Efflux From Many Adjacent Axons Influence the Excitability?

Although synchronous electrical activation of axons is unphysiological, its effect is relevant in experiments and clinical CNS stimulations using electrodes. One way to better understand the impact of such stimulation is to activate a bundle of axons and record the antidromic spike at the soma of only one of them. Then it is possible to find the axonal activation threshold of the recorded unit, obtaining both successful activations and failures with the same stimulation strength, while maintaining a relatively constant number of activated axons (Pekala et al., 2016). In such experiments, a failure at the first stimulus *increased* the failure rate at the second stimulus (changed from 50% to 75% failures at 30 ms interval), suggesting a slightly reduced excitability after a single stimulus. In contrast, a success on the first stimulus almost *eliminated* failures at the second stimulus (<0.1% failures, Pekala et al., 2016). This means that the excitability-increasing effect of a spike in the tested unit's axon was much stronger than the influence from adjacent synchronously activated axons. Similar results were reported from hippocampal CA3 cell's axons (Soleng et al., 2004).

## Shape of the DAP

The initial part of the DAP is important because, as I will explain below, evidence from grease-gap recordings and backpropagating spikes suggest that the DAP initial amplitude is close to the activation range for Na^+^ and Ca^2+^ channels. I will go through the background for those claims in the section called “How does the DAP influence axonal functions.” The start of the DAP is difficult to investigate by studying excitability (speed or threshold) because the refractory period of a spike stretches into the hyperexcitable period. A further complication is that the refractory period varies between axons (Raastad and Shepherd, 2003).

Recording the membrane potential would give information during the early period but direct intracellular voltage recordings of the traveling spike in TCAs have not been possible yet. Various methods have been used to overcome the difficulty in recording intracellularly from TCA, particularly the distal parts of the axonal arbor. These include intracellular recordings from soma with backpropagating spikes, “grease gap” methods, recordings from the bleb that forms when the axon is cut, and voltage sensitive dye (VSD) recordings. Most of these recordings have focused on the amplitude and width of the fast part of the spike, well-motivated by the importance of those parameters for control of transmitter release. Few have investigated spike after-potentials and their influence on conduction properties like speed, endurance, bursting, and failures.

**Figure 2:**
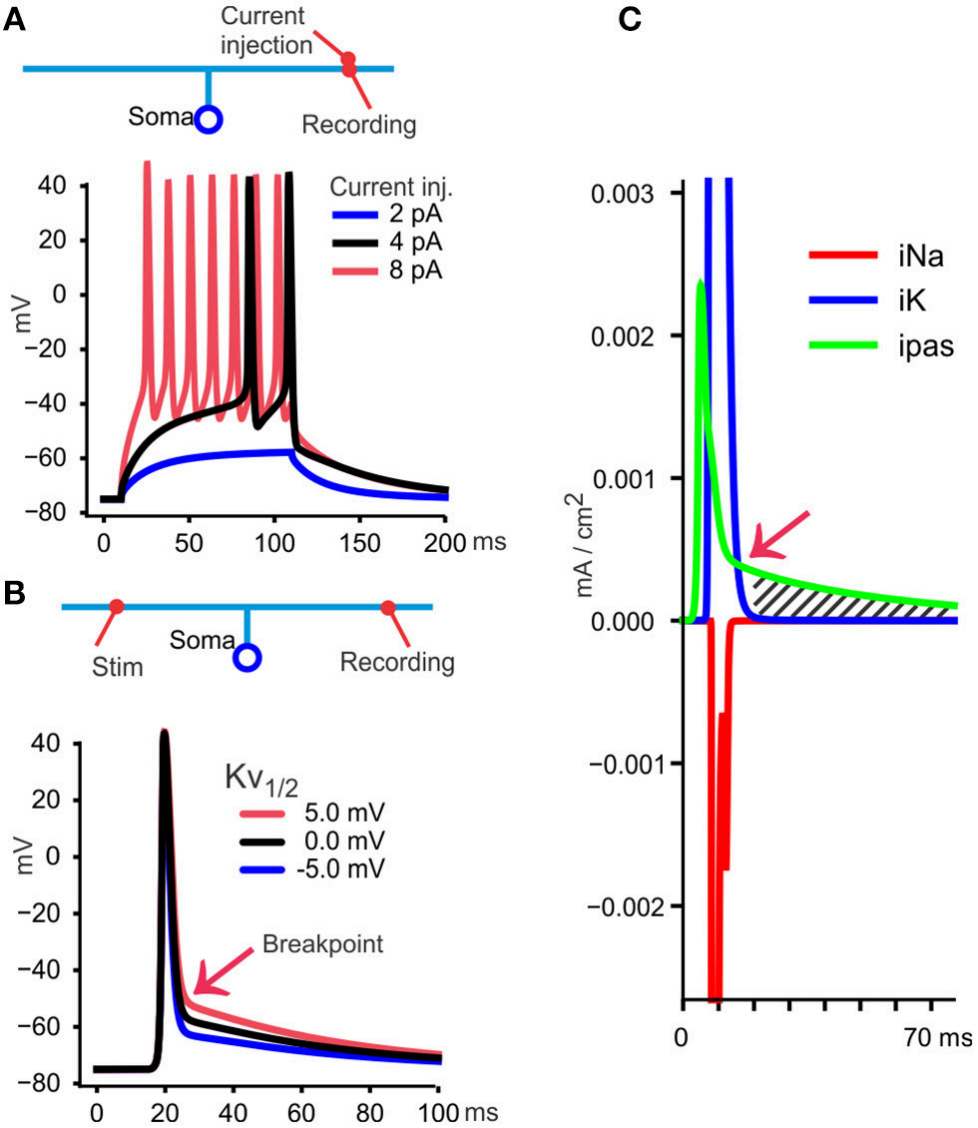
A model to illustrate essential features of the DAP and post-spike hyperexcitability. **(A)** A simple model is used to illustrate findings and interpretations from various publications. A soma with 10 μm diameter connects to a 150 μm ascending axon with 0.3 μm diameter, and divides in two branches, each 1 mm long (~3 space constants each) and 0.2 μm diameter. Those dimensions resemble a granule cell in cerebellum and have axonal diameters compatible also with Schaffer collaterals (Palay and Chan-Palay, 1974; Shepherd and Harris, 1998). The neuron is given uniformly distributed conductances and other membrane properties (see section Methods). At 30°C, commonly used in slice experiments, small currents (100 ms square pulses) give firing, measured at the site of current injection in the axon, but a DAP cannot be identified because the spikes occur from a depolarized potential relative to resting membrane potential. **(B)** When measuring the traveling spike, a DAP becomes apparent because the spike starts at a membrane potential more negative than the voltage at which the fast repolarization stops (“breakpoint,” red arrow). In this model, the breakpoint depends on the midpoint voltage for Kv's steady state activation curve (see section Methods), shown here with three values. For the rest of the simulations I used 0 mV (black curve) as midpoint. **(C)** With recording from the distal axon, as in **(B)**, the currents through Na^+^, K^+^, and passive channels (iNa, IK, ipas) are shown during the black spike in **(B)**. At one time point (red arrow) the passive current (green) becomes larger than iK and from there the DAP starts (hatched area) and follows the membrane time constant if, as here, the voltage sensitive iK is negligible.

To illustrate the findings, interpretations and qualifications, I will use a simple cable model implemented with the simulator NEURON (Hines and Carnevale, 1997). This is meant as a minimalistic model that approximately reproduce spike propagation properties discussed in this article. Details of the model are explained in Figure 2 and in section Methods.

### Backpropagating Spikes

**Figure 3:**
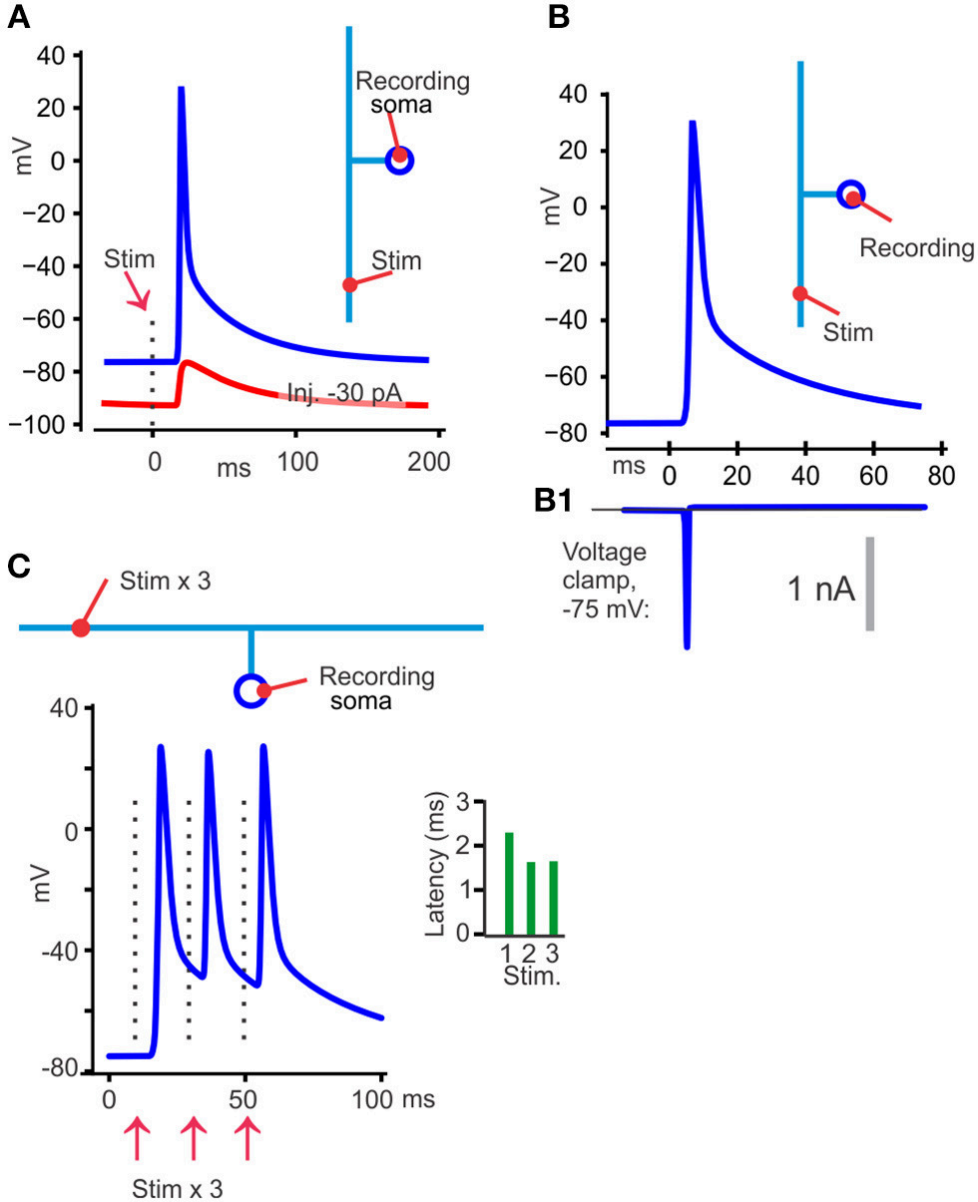
Illustration of different findings from publications showing that TCA spikes have a DAP. **(A)** With the model at its uniform resting membrane potential of −75 mV, 30°C, an axonal electrical activation resulted in a back-propagating somatic spike (blue). When the soma was hyperpolarized to −90 mV (red) with current injection, the spike failed to invade the soma, but a DAP could still be detected. **(B)** Switching from voltage recording **(B)** to voltage clamp **(B1)** at the somatic recording site eliminated the DAP, which is compatible with a passive decay and similar to the results in Palani et al. (2012). **(C)** With three stimuli 20 ms apart the latency was shorter for the second than for the first spike, but similar for the second and third (green bars). One likely explanation is that the second and third spike traveled in an axon that was depolarized to the same voltage, by the DAP, in contrast to the first spike that had to overcome a more hyperpolarized axon.

One opportunity to get information about the TCA spike, at least in neurons that have TCAs attached directly to soma without interposed myelination, like the cerebellar granule cells and rodent hippocampal Schaffer collaterals, is to use tight-seal somatic voltage recordings with backpropagating, electrically triggered spikes. This is illustrated with the model neuron in Figure 3A. Contributions from near-soma regions can be reduced or avoided by hyperpolarizing the soma by current injection to prevent the fast part of the spike from invading the soma. If there is a DAP following the axonal spike it may still be recorded at the soma, as shown in Figure 3A, red curve.

That approach (Palani et al., 2012; Pekala et al., 2014) was used to show a DAP starting on average 12 mV positive to a −63 mV resting potential and a decay to 1/e in 52 ms (Palani et al., 2012). Those publications also utilized threshold activation of the axon, with ~50% activation failures of the recorded neuron. The DAP never occurred without a spike (at somatic voltages that allowed the spikes to invade the soma), showing that the DAP is an integral part of the spike. Furthermore, at the stochastic stimulation failures there was no detectable depolarization even though many axons were activated by the constant stimulus. This means that the depolarizing effect from other axons or cells, for example by their K^+^ efflux, was not contributing much to the DAP recorded at the soma.

### Grease-Gap

“Electrical gap” methods (Stampfli, 1954), like air-, sucrose-, or grease-gap have been useful for recording membrane potential changes of axons that are hard to record from with intracellular electrodes (Stys et al., 1991). With the grease-gap method the fast decay and the much slower DAP (in parallel fibers and hippocampal Schaffer collaterals) can be distinguished (Palani et al., 2012). The time course of the DAP recorded with the grease-gap method is similar to the decay of the hyperexcitability and to the antidromic somatically recorded potential. This supports the hypothesis that the hyperexcitability is caused by a slowly decaying axonal potential. With both grease-gap and somatically recorded back-propagating spikes, the DAP starts immediately when the fast repolarization stops (see Figures 2B, 3A, blue), without any interposed hyperpolarization.

### Bleb Recordings

Although recordings from axonal blebs that form when axons are cut (Shu et al., 2007) have given very precise knowledge about spike initiation and membrane currents in the proximal 300–400 μm of axons, it has also become clear that this part of the axon is a specialized region different from more distal parts of the axons (Hu and Bean, 2018). Additionally, such recordings have been made mostly from layer 5 neocortical pyramidal cells which are myelinated (Shu et al., 2007) and relies less than the TCAs on voltage sensitive channels for fast spike repolarization (Chiu et al., 1979; Kaars and Faber, 1981). These differences in axonal mechanisms may explain that no DAP has been identified in bleb recordings.

### Voltage-Sensitive Dyes

Voltage sensitive dyes have the potential to report voltages from axons which are difficult to access with electrical recording electrodes. VSD recordings from relatively proximal parts of the TCAs, up to 200–400 μm from the soma, have not shown DAPs (Zhou et al., 2007; Foust et al., 2011; review by Popovic et al., 2015). In contrast, recordings that include distal parts of TCAs have shown apparently large DAPs (Sabatini and Regehr, 1997; Matsukawa et al., 2003). Hyperpolarizing after-potentials have not been reported with VSD recordings from TCAs, but there is one example of a small, fast repolarization that goes below the pre-spike level from cultured hippocampal neurons, using a light-activated proton pump as fluorophore (Hoppa et al., 2014).

One problem in interpreting these results is that proximal and distal VSD recordings have used different dyes and labeling methods. There is no example yet of recordings from both proximal and distal regions of TCAs that could have clarified if it is the axonal region, the recording method, or the neuron type that causes these different results. I will go through the few publications that have used VSDs to record from TCAs, and at the end suggest some factors that theoretically may influence the opportunity to record a DAP if one were present.

With bath-applied voltage sensitive fluorescent dyes (VSFD) and transmitted light voltage sensitive probes a large DAP, starting at ~30% of the amplitude of the fast spike, was recorded in rat cerebellar parallel fibers (Sabatini and Regehr, 1997; Matsukawa et al., 2003). Synaptic transmission was eliminated but [K^+^]_o_, either directly influencing membrane potential of the axons or depolarizing glia cells, were discussed as possible mechanisms. This seems unlikely because there was no addition of the DAP amplitudes at 10 ms spike intervals (Sabatini and Regehr, 1997) even though K^+^-sensitive electrode recordings show that [K^+^]_o_ ads at such intervals (Fritz and Gardner-Medwin, 1976; Malenka et al., 1981, 1983; Kocsis et al., 1983). Also, with axonally targeted genetically encoded hybrid voltage sensors there seems to be a small long-lasting DAP in mossy fibers and mossy cell axons, both unmyelinated, although this was not specifically commented or analyzed (Ma et al., 2017).

Although I focus on mammalian TCAs in this review, VSFD recordings from crayfish neuromuscular junction (Lin, 2008) are interesting because they show several features that resemble the VSFD recordings from Sabatini and Regehr (1997) and the grease-gap recordings from cerebellar parallel fibers (Palani et al., 2012). Common for these experiments is that they include the distal parts of thin, unmyelinated axons with *en passant* synapses, they have a hyperexcitable period after individual spikes (20–30% reduced activation threshold 15–25 ms after the spike, for the crayfish synapse see Zucker, 1974), and they have a slowly decaying DAP following *directly* after the fast part of the repolarization that is the likely cause of the hyperexcitability.

Because the crayfish neuromuscular synapse gives the opportunity to record presynaptically with intracellular micro-electrodes it has been extensively used to study how presynaptic mechanisms influence transmitter release. In such intra-axonal recordings, the DAP occurs only when the axon and its terminals are relatively *hyperpolarized* (Dudel, 1971). Because the occurrence of a DAP often required injection of hyperpolarizing current it has been unclear whether the DAP was a natural part of the spike in the crayfish preparation. However, since intact terminals have a large reduction of activation threshold after a spike (Zucker, 1974), and since the VSFD recordings (Lin, 2008) show a DAP, it is likely that the intact terminals do have DAPs.

These observations showing that the DAP required relatively hyperpolarized membrane potentials, and my model showing that the DAP disappears at depolarized levels (black curve in Figure 2A), give the idea that one reason some VSD recordings from TCAs do not show a DAP (in addition to the obvious reason that neocortical pyramidal neurons may not have an axonal DAP) is that the recording site was depolarized. Factors that may contribute to depolarization is that spikes were elicited by depolarizing currents at the soma (which often influences the axon at the recording site as pointed out in Foust et al., 2011), and phototoxicity. Because TCAs have a much higher surface-to-volume ratio than the soma, toxins produced by membrane-associated fluorophores will have a small cytoplasmic volume for dilution and may cause more phototoxicity in the TCA than at the soma.

## Mechanisms causing the DAP in TCAs

DAP mechanisms have been investigated at the soma, dendrites, large presynaptic terminals, and axons. Inward currents (Na^+^ and Ca^2+^) and passive components have been identified. For example, in hippocampal CA1 pyramidal neurons voltage sensitive R-type Ca^2+^ channels are important (Metz et al., 2005); in cerebellar granule cells a resurgent Na^+^ current contribute (Magistretti et al., 2006). In presynaptic structures large enough for intracellular recordings a DAP has been found in calyx of Held (Borst et al., 1995), and in mossy fiber boutons (Geiger and Jonas, 2000). In these terminals the DAP has both passive and active components (Kim et al., 2010; Lewis and Raman, 2014; Ohura and Kamiya, 2018). Myelinated axons usually have a passive DAP (Barrett and Barrett, 1982). The high resistance between the myelin sheet and the axonal membrane gives the DAP its slow decay and this mechanism is therefore unlikely to contribute in unmyelinated TCAs.

As I will explain in the following sections there is no evidence that inward currents contribute to the DAP in cerebellar parallel fibers or Schaffer collaterals. That does not mean that inward currents may not influence the DAP under conditions not yet tested, for example during longer spike trains. In the lack of data showing effects that need inward currents to be explained, I will exclude further discussions about the contribution of inward currents in other cells, presynaptic structures, or soma.

I will distinguish between mechanisms that determine the decay rate and those that determine the amplitude in TCAs. For all experiments referred in the three following sections, fast synaptic currents are blocked by CNQX, APV, and Picrotoxin. Under such block the shape of the DAP did not change with the addition of 200 μM Cd^2+^, showing that neither release nor voltage sensitive Ca^2+^ currents contributed (Toda, 1976).

### DAP Decay in TCAs

Three observations suggest that the decay of the DAP is passive, meaning determined by the membrane capacitance and resistance. First, when the antidromic spike with its DAP is recorded at the granule cell's soma, and the recording is switched to voltage clamp, there is no detectable current that can explain the DAP, although a significant fast unclamped spike current persisted (Palani et al., 2012). This is expected from a capacitive current and can be illustrated with the model (somatic voltage clamp simulation in Figures 3B,B1).

Second, the decay rate of the post-spike hyperexcitability is not more than 20–30% different at 24 and 34°C (Soleng et al., 2004). This is compatible with the temperature effect on the membrane time constants of several cell types (Kim and Connors, 2012) but does not rule out other mechanisms with low temperature sensitivity.

Third, spike activity that probably induce changes in membrane resistance change the decay rate of the hyperexcitability (Soleng et al., 2004). The background for that experiment is that the Na-K-pump is electrogenic and hyperpolarizes the membrane as it pumps out excess Na^+^ (Ritchie and Straub, 1957). In many axons this hyperpolarization is reduced by opening of HCN-channels, underlying the H-current (Eng et al., 1990). This added conductance (reduced membrane resistance) will reduce the membrane time constant. So, when the post-spike hyperexcitability decays ~3 times faster at 2 Hz spike frequency compared to 0.1 Hz and becomes ~2 times slower at those frequencies when HCN channels are blocked, a likely explanation is that an HCN conductance reduces the membrane resistance and the membrane time constant and therefore gives a faster DAP decay (Soleng et al., 2004).

### Amplitude of the TCA's DAP

The mechanisms governing the DAP amplitude has been explored by studying how it is influenced by activity, changes in membrane potential, and drugs (next section).

Several lines of evidence suggest that the voltage at which the fast repolarization stops, and the DAP starts (the “breakpoint” in Figure 2B) is relatively constant and insensitive to pre-spike potential, and spike activity. Voltage sensitive channels that inactivate fast at relatively depolarized levels, like the channel mechanism I implemented in the model, could explain these properties of the breakpoint (Figure 3C). The evidence for the breakpoint's insensitivity to pre-spike potential and spike activity comes from experiments similar to those referred under “Shape of the DAP” above but includes trains of axonal spikes. The constant, depolarized voltage for the breakpoint during such trains (illustrated in Figure 3C) has been observed by using VSFD, transmitted-light voltage sensitive probes, grease-gap recordings and somatic recordings with backpropagating spikes in cerebellar parallel fibers (Sabatini and Regehr, 1997; Matsukawa et al., 2003; Pekala et al., 2016). Those publications show that the second spike in such trains take off from the “back” of the DAP, a potential more depolarized than the resting membrane potential, but this difference in pre-spike voltage does not influence the breakpoint. The DAP also gives the spikes after the first one faster conduction speed, as seen in the green histogram in Figure 3C.

Those recordings from cerebellar parallel fibers are, with respect to constant breakpoint, very similar to recordings from the crayfish neuromuscular junction (Lin, 2008). A repolarization voltage relatively insensitive to pre-spike potential was also shown *in vivo* in the calyx of Held (Sierksma and Borst, 2017). Like spikes have a narrow voltage range for being triggered at the AIS, there may be a similarly narrow range for the end of their fast repolarization at the presynaptic structures in several neuron types and several species.

### Channels

The channels responsible for the fast repolarization which, according to data discussed above, also determine the DAP amplitude (measured from baseline to the breakpoint) have been explored by bath-applied drugs during grease-gap recordings (Pekala et al., 2014). The spike in cerebellar parallel fibers was not sensitive to 200 μM Cd^2+^, ruling out Ca^+^ currents and Ca^+^- activated currents. Relative to baseline, the breakpoint moved 10% in depolarizing direction in response to 1 mM TEA, and 27% in response to 10 nM margatoxin (MgTX), when these drugs were applied alone. However, in combination these drugs took away most of the fast repolarization by moving the breakpoint 75% in depolarizing direction. The interpretation is that both MgTX-sensitive and TEA-sensitive channels contributed to the fast repolarization that stopped at the breakpoint and did a better job together than alone.

To interpret such grease-gap experiments it is important to be aware that by measuring the difference between baseline and the breakpoint we cannot, without further control experiments, know if it is the baseline (corresponding to resting membrane potential) or the breakpoint voltage that changes. Two additional observations help: First, conduction speed is sensitive to resting membrane potential, and no change was detected with either Cd^2+^, TEA, or MgTX (Pekala et al., 2014). Second, MgTX enhanced the post-spike *hyperexcitability* (Palani et al., 2010), supporting the interpretation that TEA and MgTX pushed the breakpoint and the decay of the DAP in depolarizing direction.

Low concentrations of TEA (≤ 1 mM) have preferential effect on Kv3-family of channels (Rudy and McBain, 2001) and because Kv3.1 and Kv3.3 double knockout mice (Matsukawa et al., 2003) lack most TEA effects on cerebellar parallel fibers, these channels are likely to mediate the TEA effects at least in those axons. MgTX blocks Kv1.3 channels with high affinity, although others in the Kv1 family may also be blocked.

## How Does the DAP Influence Axonal Functions?

The data referred above suggest that the DAP is a robust process, starting at a voltage controlled by more than one channel family, relatively insensitive to activity and temperature, with a decay determined mainly by the membrane time constant. How does a spike with those properties influence axonal function?

### The DAP Increases Reliability

The common view that TCAs never fail under physiological conditions may be biased by few rodent experiments using temperatures above 35–36°C, while rodents, including their brains, often raise above 38°C during moderate activity (Kiyatkin et al., 2002). Temperature dependent failures can be demonstrated by measuring populations of spikes at two sites along parallel fibers in cerebellum. When temperature increases just above 37°C the fraction of spikes arriving at the distal electrode falls (Pekala et al., 2016). VSDF recordings have also shown reduced spread of signals in hippocampal slices at temperatures >37°C (Takeya et al., 2002). Failures are provoked by higher temperature because Nav inactivation and Kv activation become faster (Hodgkin and Katz, 1949), both factors that reduce the spike amplitude and integral. Particularly fast activating Kv3 channels, present in Schaffer collaterals and cerebellar parallel fibers, would theoretically increase failure probability at high temperatures.

**Figure 4:**
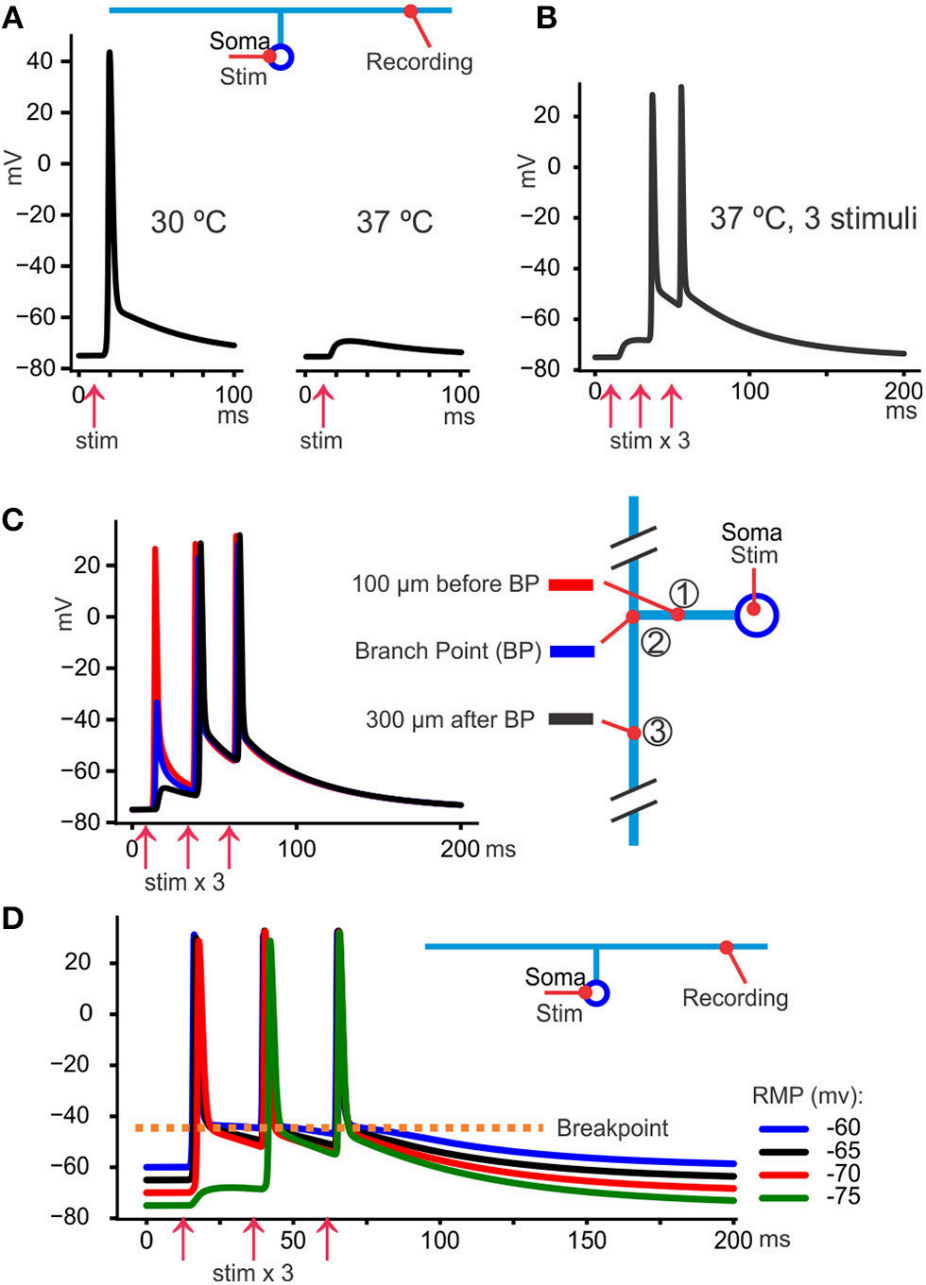
The DAP reduces propagation failures. **(A)** At 30°C the spikes triggered at the soma by current injection always arrived at the axon. However, at 37°C they were still triggered at the soma (not shown) but failed to arrive at the distal axon where only a small voltage hump was seen. **(B)** Keeping the temperature at 37°C it was possible to overcome the propagation block by repeating the somatically triggered spikes, in this case with 20 ms intervals. One likely explanation is that the slow DAP spread passively further than the fast part of the spike (as can be seen as a hump in response to the first stimulus) and facilitated the propagation of the second and third spike. **(C)** By recording from three sites in the model axonal arbor we see that the first spike failed to propagate between site 1 and 3, but a depolarizing hump, due to the DAP, propagates even 300 μm past the branch point. That residual depolarization helped the second and third spike to propagate further than the first. **(D)** The resting membrane potential (RMP) was adjusted by changing the extracellular K^+^ concentration to obtain values between −60 and −75 mV (color coded). We see that the fast-deactivating K^+^ conductance implemented in the model gave very similar voltage levels for the breakpoint despite the large variation in RMP.

When spike propagation failures are provoked by temperature (illustrated with the model in Figure 4A), propagation reliability increases with the number of stimuli (Figures 4B,C). With somatic recordings and antidromic spikes some spikes occur only as a hump, but the next spike usually succeeds to propagate all the way to the soma (Pekala et al., 2016). This suggests that the post-spike hyperexcitability, due to the DAP, reduces the failures toward the end of the spike train. The proper test of that interpretation would be to move the “breakpoint” in hyperpolarizing direction to reduce its effect on failures. This has not been tested yet but may be possible using pharmacological tools (Kaczmarek and Zhang, 2017).

### Ca-Tail

Almost every bouton, which is one of the structures that defines a TCA, has machinery for transmitter release, including voltage sensitive Ca^2+^ channels. This makes the shape of the *propagating* spike important for transmitter release and a broad range of normal and pathological processes (Debanne et al., 2011; Fioravante and Regehr, 2011). Since subthreshold potentials between −60 mV and spike threshold have been shown to give asynchronous transmitter release (in crayfish neuromuscular junction: Wojtowicz and Atwood, 1984) and to open Ca^2+^ channels (mouse calyx of Held: (Awatramani et al., 2005); rat cerebellar interneurons: Christie et al., 2011) there is a theoretical possibility that the DAP may increase presynaptic Ca^2+^ or slow the deactivation of Cav channels. This may contribute to the early phase of the delayed transmitter release occurring at some synapses, for example between parallel fibers and Purkinje cells (Atluri and Regehr, 1998). Whether Cav channels activate or not may also depend on a finetuned control between membrane potentials immediately before the spike and the amplitude of the DAP (Clarke et al., 2016), suggesting that DAP amplitude may be decisive in such delayed transmitter release.

### Role in Bursting

It is not known if axonal (ectopic) bursts occur *in vivo*, but in slice experiments axonal bursts occurs in ~30% of cerebellar granule cells. Such bursts come on the shoulder of the backpropagating spike's DAP, suggesting that the DAP is close to spike threshold (Pekala et al., 2014).

In the context of axonal bursting the temporal overlap between the refractory period (inhibiting bursting) and the DAP (promoting bursting) may be important. Since the post-spike hyperexcitability is relatively insensitive to temperature (Soleng et al., 2004), but the refractory period is very temperature sensitive (Raastad and Shepherd, 2003), one hypothesis is that bursts occur with higher probability at high temperature because the Nav-channel inactivation becomes too short-lasting to protect against new spikes while the DAP is at its most depolarized levels. Theoretically, this could contribute to epileptic temperature-sensitive pathology, but that hypothesis has not been tested yet.

The stable voltage at which the fast repolarization stops has been shown to stabilize the shape of the spike during bursts, in crayfish neuromuscular junction (Lin, 2008) and in mouse calyx of Held (Sierksma and Borst, 2017). This may be important to minimize energy expenditure, and control transmitter release.

The constant breakpoint voltage will also, theoretically, stabilize the spike shape in situation where [K^+^]_o_ increases, particularly relevant in pathology like epileptic seizures. Figure 4D illustrates how the model behaves at different RMPs, manipulated by changing [K^+^]_o_. We see that during a train of spikes the individual spikes take off from similar potential despite great differences in RMPs, protecting the spiking mechanism from variations in [K^+^]_o_.

## Conclusion

The main conclusion of this article is that there is good support for the hypothesis proposing that individual spikes in cerebellar parallel fibers and hippocampal Schaffer collaterals are followed by a DAP, and that the DAP is a well-controlled process that stops the fast repolarization at a membrane potential positive to resting membrane potential, leaving the membrane more excitable for ~100 ms during a slow, passive discharge of the membrane capacitance.

It is important that the presented model is intentionally simple but still qualitatively compatible with the selected data. The data I have selected is related to excitability, like activation threshold, conduction speed, and propagation failures. There is much data for example on transmitter release properties that the model is not intended to explain. The excitability control of the TCAs is probably enormously more complex, with interactions between changes in intracellular ion concentrations, the Na-K-pump, structural inhomogeneities along the TCAs, and participation of a large number of ion channels.

## Methods

### Neuronal Model

Two voltage sensitive channels, Nav and Kv, and a leak conductance (gL) were used for the cell model. Both were implemented with the NMODL and NEURON languages (Hines and Carnevale, 1997) and inserted uniformly in the membrane of a neuron with dimensions given in Figure 1. Membrane capacitance and cytoplasmic resistivity were set to 1 μF/cm^2^ and 150 Ωcm. The Nav had one variable for activation (m) and one for inactivation (h), and the Kv had one for activation (n), like the Hodgkin-Huxley (HH) model (Hodgkin and Huxley, 1952):

$$\begin{array}{l} \text{I}=C\cdot \frac{dV}{dt}+\text{gK}\cdot n^{4}\cdot \left(V_{m}-V_{K}\right)+\text{gNa}\cdot m^{3}\cdot \text{h}\cdot \left(V_{m}-V_{Na}\right)+\text{gL}\left(V_{m}-V_{L}\right) \end{array}$$

The gating variables were calculated as $\frac{dx}{dt}=\left(\frac{1}{xTau}\right)\;\cdot \;\left(xInf-x_{0}\right)$, where x is n, m or h.

The values of xInf was calculated as $xInf=\frac{1.0}{\left(1.0+\exp\left(\frac{v1}{-steepness}\right)\right)}$, and xTau as $xTau=\frac{Tmax}{qt}\;\cdot \;\left(0.05\;+\;\left(\frac{1.9}{exp\left(\frac{v1}{Tc}\right)+exp\left(-\frac{v1}{Tc}\right)}\right)\right)$, using $qt=2.3^{\frac{celcius-23}{10}}$, *v*1 = *v* − *midpoint*.

The “midpoint” for n, m, and h was 0, −25, −40. The “steepness” for n, m, and h was 18, 6, and 3. The “Tc” for n, m, and h was 25, 20, 10, and the “Tmax” for n, m, and h was 15, 0.51, and 7. Like HH, the conductance at each time step (0.01 ms) was calculated as gmax ^*^ n^4^ for Kv, and gmax ^*^ m^3^^*^h for Nav, with gmax (the maximal conductance) 0.05 and 0.08 S/cm^2^ for Nav and Kv, respectively. The parameters were chosen by manually selecting values that gave a fast Na^+^ and K^+^ current, and a fast deactivation of the K^+^ current with the parameter “midpoint” set to a sufficiently depolarized value (0 mV) to give an almost complete deactivation at −45 mV.

The gL was set to 0.00002 S/cm^2^ (which gives a 50 ms membrane time constant, compatible with the decay of the DAP in grease-gap recordings in Palani et al., 2012). Equilibrium potentials were 50 mV for Nav, −85 mV for Kv, and −75 mV for gL, which are common values in the literature.

## Author Contributions

The author confirms being the sole contributor of this work and has approved it for publication.

### Conflict of Interest Statement

The author declares that the research was conducted in the absence of any commercial or financial relationships that could be construed as a potential conflict of interest.
